# Dry Etching Characteristics of InGaZnO Thin Films Under Inductively Coupled Plasma–Reactive-Ion Etching with Hydrochloride and Argon Gas Mixture

**DOI:** 10.3390/ma17246241

**Published:** 2024-12-20

**Authors:** Changyong Oh, Myeong Woo Ju, Hojun Jeong, Jun Ho Song, Bo Sung Kim, Dae Gyu Lee, ChoongHo Cho

**Affiliations:** 1 DRAM Process Architecture Team, Samsung Electronics, Hwaseong-si 18448, Gyeonggi-do, Republic of Korea; cy2086.oh@samsung.com; 2 Department of Applied Physics, Korea University, Sejong 30019, Republic of Korea; skoks@korea.ac.kr; 3 E ICT-Culture Sports Track, Korea University, Sejong 30019, Republic of Korea; 4 Division of Display and Semiconductor Physics, Korea University, Sejong 30019, Republic of Korea; hojun0131@korea.ac.kr (H.J.); jhsong@korea.ac.kr (J.H.S.); 5 Department of Computer Information Science, Korea University, Sejong 30019, Republic of Korea; chcho@korea.ac.kr

**Keywords:** InGaZnO (IGZO), dry etching, inductively coupled plasma–reactive-ion etching (ICP-RIE), thin-film transistor, non-fluorine-based etching gas

## Abstract

Inductively coupled plasma–reactive etching (ICP-RIE) of InGaZnO (IGZO) thin films was studied with variations in gas mixtures of hydrochloride (HCl) and argon (Ar). The dry etching characteristics of the IGZO films were investigated according to radiofrequency bias power, gas mixing ratio, and chamber pressure. The IGZO film showed an excellent etch rate of 83.2 nm/min from an optimized etching condition such as a plasma power of 100 W, process pressure of 3 mTorr, and HCl ratio of 75% (HCl:Ar at 30 sccm:10 sccm). In addition, this ICP-RIE etching condition with a high HCl composition ratio at a moderate RIE power of 100 W showed a low etched pattern skew and low photoresist damage on the IGZO patterns. It also provided excellent surface morphology of the SiO_2_ film underneath after the entire dry etching of the IGZO layer. The IGZO thin film as an active layer was successfully patterned under the ICP-RIE dry etching under the HCl-Ar gas mixture, affording an excellent electrical characteristic in the resultant top-gate IGZO thin-film transistor.

## 1. Introduction

Amorphous oxide semiconductors have attracted much attention as active materials of switching and driving devices in the thin film transistor (TFT) backplane of organic light-emitting diode displays because oxide semiconductor-based TFTs have low off-current, excellent field-effect mobility, low subthreshold slope, and good current uniformity [1,2,3,4,5]. Among many candidates of oxide semiconductors, InGaZnO (IGZO) has been widely used in active matrix displays, integrated circuits, photodetectors, and future flexible and wearable devices [6,7,8,9,10]. The recent increase in the applicability of ultra-high-resolution displays to augmented reality and virtual reality areas requires a much smaller feature size for TFTs in the display pixels [11,12,13,14,15]. Photolithography and etching are the main processes for scaling TFTs. While the conventional IGZO patterning method is well-established based on wet etching, dry etching for IGZO patterning has not been sufficiently studied [16,17,18,19,20]. Wet etching methods using chemical solutions for IGZO etching have fast etching speed and good selectivity. However, they are unsuitable for fine pattern formation because of their isotropic etching behavior [21,22]. As usual, plasma etching processes are favorable for achieving fine patterns with high aspect ratios owing to their anisotropic etching characteristics. Studies on the etch of IGZO in ICP-RIE mode have used etching gases, including several fluorine or chlorine atoms in the molecule, such as CF_4_, SF_6_, or BCl_3_, to increase the etching performance [16,17,18,19]. Since these gases cause environmental problems such as global warming, the semiconductor and display industries have recently tended to avoid their use. Recently, we conducted a study on an eco-friendly dry IGZO etching process under capacitively coupled plasma–reactive-ion etching (CCP-RIE) mode using hydrochloride (HCl) and argon (Ar) gases that do not contain perfluorocarbons (PFC) as etching gases commonly used in dry etching of oxide thin films [23]. However, CCP-RIE had the disadvantage of a slow etch rate of about 6 nm/min for IGZO film due to the low plasma density.

In this paper, an inductively coupled plasma–reactive-ion etching (ICP-RIE) of an IGZO thin film was studied to enhance the etching rate of the IGZO with high plasma density using HCl and Ar mixture as etching gases without perchloro- or perfluoro-etching gases, while minimizing the etching damage of photoresist (PR) and the surface of SiO_2_ film underneath after the entire dry etching of the IGZO layer. Dry etching characteristics were investigated for radio-frequency (RF) bias power, chamber working pressure, and etching gas composition ratio as process parameters. In addition, etching conditions for increasing the selectivity with the SiO_2_ layer under IGZO films were studied.

## 2. Materials and Methods

IGZO film deposition, etching, and analysis: IGZO (70 nm) were sputter-deposited on 6-inch dry-oxidized (SiO_2_) Si wafer (SK Siltron) substrates under an RF power of 50 W and a working pressure of 5 mTorr with a gas mixture of Ar (95 sccm) and O_2_ (5 sccm) at room temperature using an IGZO target (atomic composition of In:Ga:Zn = 1:1:1). For dry etching of IGZO thin films, an ICP-RIE reactor (KEVT-i2000L, Korean Vacuum Tech, Gimpo-si, Republic of Korea) containing an inductively coupled plasma coil device for the generation of high plasma density was used along with an RF-powered capacitively coupled plasma (CCP) capacitor device operating at 13.56 MHz. Flow rates of etching gases were adjusted using mass flow controllers (G-Series GE50A, MKS Instrument, Andover, MA, USA). IGZO etch rates were examined under different working pressures, HCl/(HCl + Ar) composition ratios, and RIE bias power. The etch rates were calculated by measuring the thickness of IGZO and SiO_2_ films before and after etching using an ellipsometer (SEMG-1000, Nano-view, Ansan, Republic of Korea). The surface morphologies of IGZO and SiO_2_ films were measured using an atomic force microscopy (AFM) system (XE-100, Park Systems, Suwon, Republic of Korea). IGZO-etched pattern images and 2-dimensional depth profiles were measured using a laser microscope (VK-X3000, Keyence, Osaka, Japan). Focused ion beam–transmission electron microscopy (FIB-TEM; Helios 460F1, FEI, Lausanne, Switzerland) analysis was performed to observe the cross-section of the etched pattern of IGZO. The etching selectivity of IGZO/SiO_2_ and IGZO/PR was calculated from the following Equations (1) and (2); for example, when the etch rates of IGZO and SiO_2_ are 83.2 nm/min and 59.4 nm/min, respectively, under the etching condition of plasma power of 100 W, process pressure of 3 mTorr, and HCl ratio of 75%, the IGZO/SiO_2_ etching selectivity is 1.40.

(1) $$Selectivity\;of\;IGZO/{SiO}_{2}=\frac{etch\;rate\;of\;IGZO}{etch\;rate\;of\;{SiO}_{2}}$$

(2) $$Selectivity\;of\;IGZO/PR=\frac{etch\;rate\;of\;IGZO}{etch\;rate\;of\;PR}\;$$

Top-gate IGZO TFT device fabrication: Indium Tin Oxide (ITO; 20 nm) was deposited and patterned to form source and drain (S/D) electrodes on the glass substrate. The IGZO (30 nm) was then sputter-deposited. The IGZO films were photo-patterned and dry-etched under ICP-RIE mode, bias power of 100 W, and working pressure of 3 mTorr with HCl and Ar gas mixture (HCl:Ar = 30 sccm:10 sccm). Al_2_O_3_ as a gate insulator layer was deposited by plasma-enhanced atomic layer deposition (PEALD) using trimethyl aluminum (TMA) as a metal source and O_2_ as a reactant. Plasma power was set to be 100 W at 250 °C. One cycle of atomic layer deposition (ALD) was performed with the following sequence: TMA source injection: (0.5 s)–argon (Ar) purge (3.0 s)–O_2_ injection (1.0 s)–RF-plasma (0.5 s)–Ar purge (5 s). The thickness of the Al_2_O_3_ layers was set to 10 nm. After S/D contact holes were formed on the Al_2_O_3_ dielectric layer by photolithography, the Mo (50 nm) gate electrode was sputter-deposited and patterned to afford IGZO TFTs with a top-gate staggered configuration. The IGZO TFT was post-annealed at 350 °C for 1 h. Current-voltage (I–V) characteristics of the TFT were then measured with gate voltage (V_g_) sweep of −2 V to +2 V at a constant drain voltage (V_d_) of +0.1 V and +1 V, respectively, using a semiconductor device analyzer (B1500A, Keysight) at room temperature under dark air ambient condition.

## 3. Experimental Section

**Figure 1:**
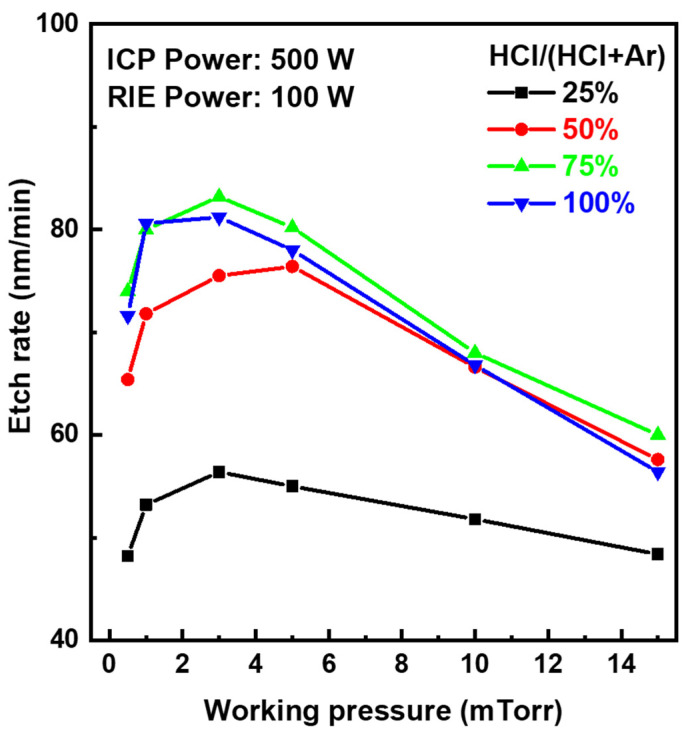
Etch rates of the IGZO films as a function of the gas mixing ratio of HCl/(HCl + Ar) according to the working pressure in the ICP-RIE process chamber at a source power of 500 W and bias power of 100 W.

**Figure 2:**
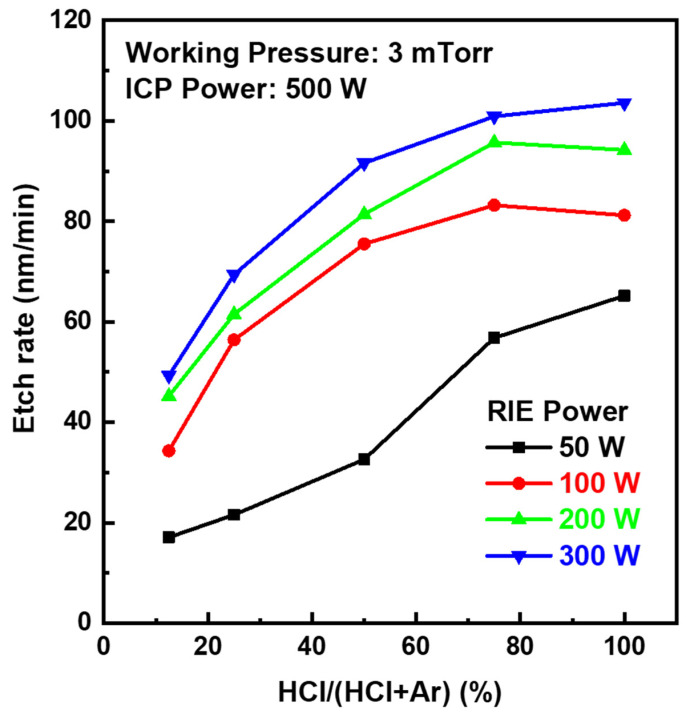
Etch rates of the IGZO films as a function of the gas composition ratio of HCl/(HCl + Ar) according to the bias power from 50 W to 300 W at a process pressure of 3 mTorr and source power of 500 W.

Dry etching characteristics of IGZO films were investigated with different HCl and Ar gas mixing ratios and process parameters, such as working pressure and bias power in the ICP-RIE chamber. Figure 1 shows the etch rates of the IGZO films as a function of the gas mixing ratio of HCl/(HCl + Ar) according to the process pressure at a source power of 500 W. When HCl increased from 25% to 50%, the etch rate of IGZO increased rapidly. The highest etch rate was found in chamber pressure of 3 mTorr at an HCl composition ratio of 75% (HCl:Ar = 30 sccm:10 sccm). The sharp decrease in the etch rate of IGZO at the working pressure below 3 mTorr might be due to the reduction in the density of etching ions. At the working pressure above 3 mTorr, the etch rate gradually decreased. This might be attributed to the decrease in the kinetic energy of etching species, as the mean free paths of the chlorine (Cl) and hydrogen (H) active ion species became shorter at higher working pressure. When the working pressure exceeds the critical pressure of 3 mTorr, the kinetic energy of the active ion species will decrease by collision before they reach the IGZO film surface. The decreased mean free path of the species could have reduced the IGZO etch rate. The maximum etch rate of the IGZO was 83.2 nm/min at a bias power of 100 W, 3 mTorr, and the HCl composition ratio of 75%. The etch rate could have been accelerated by the physical bombardment of ions or radicals with high plasma density generated from high ICP source power of 500 W. Figure 2 shows the etch rates of the IGZO films as a function of the gas composition ratio of HCl/(HCl + Ar) according to the bias power at a process pressure of 3 mTorr. As the HCl gas ratio increased, the IGZO etch rate increased. Boiling points (b.p.) of metal halides such as GaCl_3_ (b.p. 201 °C), ZnCl_2_ (b.p. 732 °C), and InCl_3_ (b.p. 600 °C) produced from etching reactions of IGZO with chlorine-type gases are relatively low compared to those of corresponding metal oxides such as Ga_2_O_3_ (b.p. 2204 °C), ZnO (b.p. 2360 °C), and In_2_O_3_ (b.p. n/a) [24]. Thus, the etching process on metal oxides would be accelerated by the easy sublimation of chlorinated by-products. A few have reported IGZO dry etching under chlorine-based plasma with Cl_2_-Ar, BCl_3_-Ar, and BCl_3_ [17,18,19,20]. We used the HCl-Ar gas mixture as a chlorine-based etching gas for the dry etching of IGZO. Although HCl is a dry etching source containing only one Cl atom in the gas molecule, it is comparable to the conventional multi-chlorine chemical species in terms of the etch rate of the IGZO film. In the case of plasma etching conditions of HCl/(HCl + Ar) of 75–100%, the IGZO etch rate was similar to that of Cl_2_-Ar plasma (50–70 nm/min at RF power of 700 W, dc bias voltage of 200 V, the gas pressure of 5 mTorr) [20]. In addition, as the bias power in the HCl-Ar plasma increased, the etch rate of the IGZO film increased. The etch rate rapidly increased to 70 nm/min at the HCl composition ratio above 75% and a bias power above 100 W. However, the IGZO etch rate was significantly lowered to less than 20 nm/min on the HCl composition ratio below 50% and the bias power of 50 W. This meant that both chemical etching with reactive Cl ions and physical etching by sputtering on high bias power had a significant impact. As the bias power applied to the blocking capacitance of CCP enhanced the self-bias, the high DC bias induced at the substrate increased the etch rate of the IGZO films. These enhancements of the IGZO etch rate could be attributed to the physical bombardment of the active species with the high kinetic energy due to the increased DC bias from high RF source power and the chemical etching from the high-density ICP plasma species, including chlorine, hydrogen, and Ar ions on the IGZO surface. We especially noted the role of hydrogen in HCl gas from the results of a high etch rate, even though HCl is the etching gas having one reactive chlorine atom. HCl shows a plasma chemistry similar to the gas mixture of Cl_2_ + H_2_ because HCl is dissociated under plasma conditions to provide chlorine and hydrogen ions at a ratio of 1:1. Hydrogen ions can create volatile etch products such as hydroxy group by chemical reaction with oxides on metal oxide surface, which helps break metal-oxygen bonds on surface oxides as seen in the ITO dry etch case [25,26,27]. Therefore, the hydrogenation on the surface of IGZO may also boost plasma reactivity between reactive chlorine ions and metals.

## 4. Analysis and Discussion

**Figure 3:**
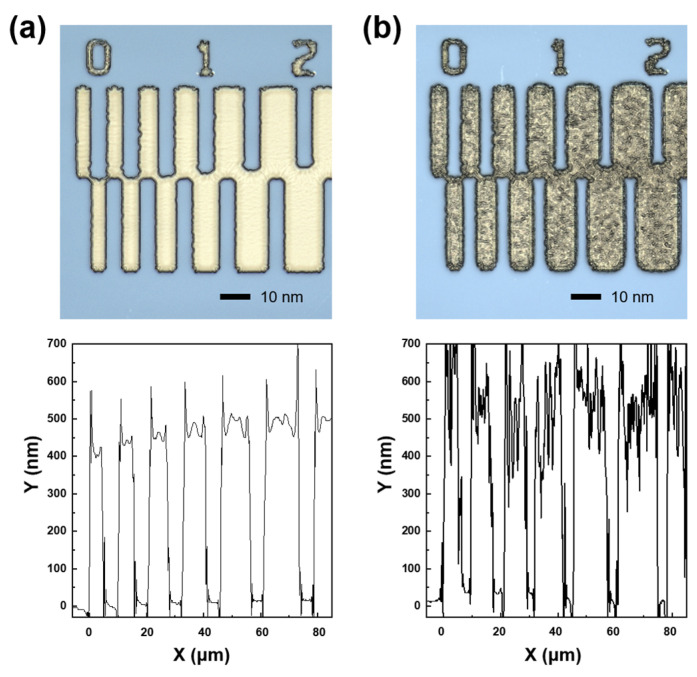
Microscopic pattern images and 2-dimensional depth profiles after IGZO dry-etching at bias power of (**a**) 100 W and (**b**) 200 W.

**Figure 4:**
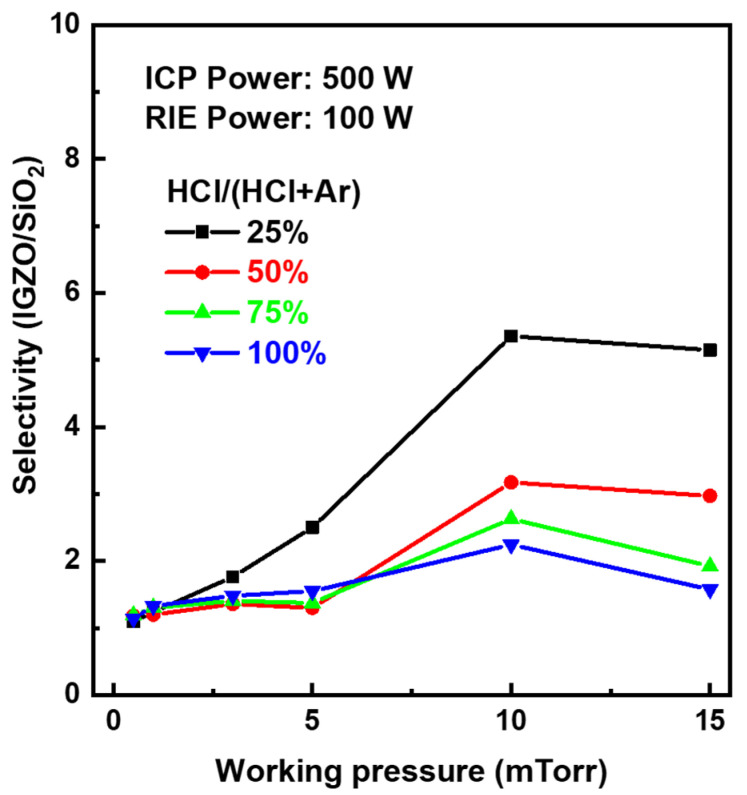
Etching selectivity of IGZO/SiO_2_ as a function of the HCl composition ratio according to the process chamber working pressure at the ICP power of 500 W and bias power of 100 W.

**Figure 5:**
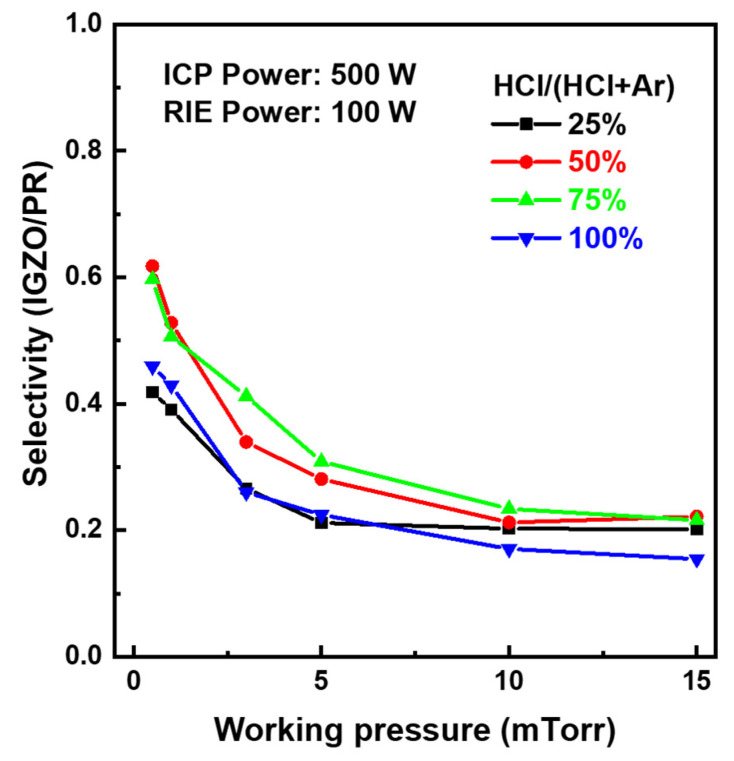
Etching selectivity of IGZO/PR as a function of HCl/(HCl + Ar) ratio according to process pressure at source power 500 W and bias power 100 W.

**Figure 6:**
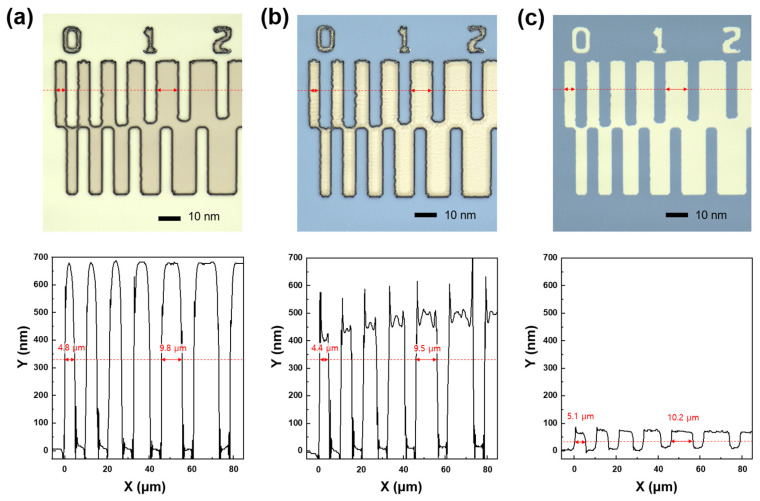
Microscopic pattern images (**top**) and 2-dimensional depth profiles (**bottom**) (**a**) after photo-process, (**b**) after IGZO dry-etching, and (**c**) after PR strip. The red dotted lines are where pattern widths were measured.

**Figure 7:**
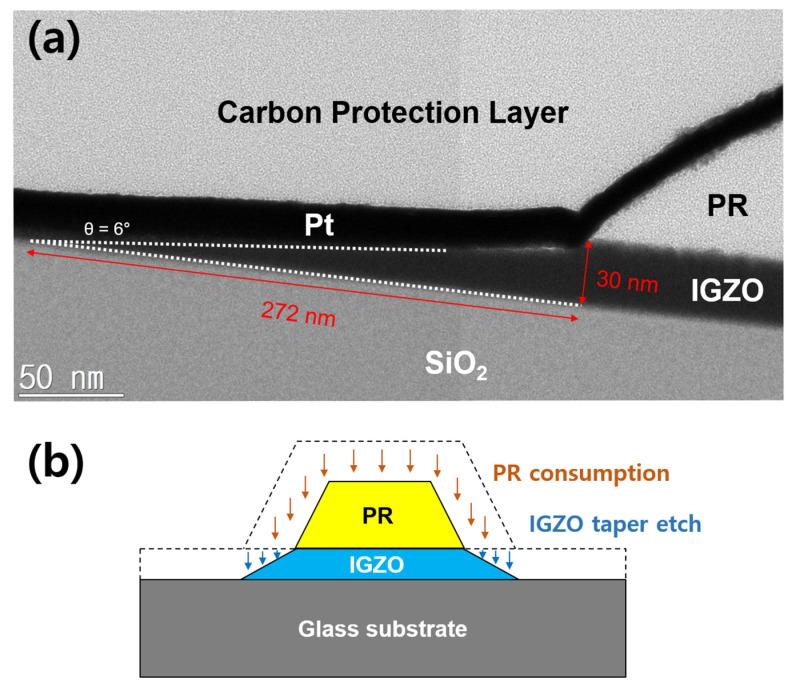
(**a**) FIB-TEM image of IGZO etch profile with an excellent taper angle of 6 degrees under PR. (**b**) Schematic diagram of taper etching mechanism of the IGZO film under ICP-RIE mode.

On the other hand, etching characteristics of materials involved in the IGZO dry etching process, such as PR and SiO_2_ layers, were investigated to figure out an optimal condition in the etching process window of around 75% HCl and 100 W bias power rapidly increasing IGZO etch rates. Figure 3a,b show the pattern images and depth profiles after IGZO dry-etching at bias power 100 W and 200 W, respectively. After the IGZO film was fully etched at the bias power of 100 W, the surface of PR was clean. However, the PR surface in the bias power of 200 W was severely damaged, as shown in the top microscope image of Figure 3b, which caused a problem like PR burning [28]. The PR pattern edges were also round-shaped at 200 W. As shown in two-dimensional depth profiles by a laser microscope at the bottom of Figure 3, when the bias power was applied too large during the dry etch process, the top surface of the PR became severely rough. In addition, PR swelled at 200 W, increasing the pattern width. Therefore, we tried to secure the optimal IGZO etch condition by ICP dry-etching within the range of not exceeding the bias power of 100 W for PR damage-free. Meanwhile, the etching characteristics of SiO_2_ under a series of ICP-RIE conditions showed a similar trend to that of IGZO. Figure S1 shows the SiO_2_ etch rates as a function of the HCl composition ratio according to the chamber working pressure at the ICP source power of 500 W and bias power of 100 W. The etch rate of SiO_2_ decreased as the working pressure increased and rapidly increased in the HCl ratio above 50%. Figure 4 shows the etching selectivity of IGZO/SiO_2_ as a function of the HCl composition ratio according to the process chamber working pressure at the bias power of 100 W. IGZO/SiO_2_ selectivity increased with increasing working pressure. However, the selectivity of IGZO/SiO_2_ in the HCl composition ratio of less than 25% would not be meaningful owing to the low etch rate of the IGZO, as shown in Figure 1 and Figure 2. The selectivity was not significantly different according to the working pressure at an HCl composition ratio of 75% or more, and the selectivity exhibited above 1.0 even at a low pressure of 5 mTorr with high IGZO dry-etching rates. The PR etch rate was also examined as a function of the HCl composition ratio according to the chamber working pressure at the ICP source power of 500 W and bias power of 100 W. The etch rate of the PR was much faster than that of IGZO and SiO_2_. As shown in Figure S2, the etch rate ranged from 200 to 400 nm/min at the working pressure above 3 mTorr. The etch rate was significantly increased under 100% HCl condition. Figure 5 shows IGZO/PR selectivity as a function of the HCl/(HCl + Ar) ratio according to process pressure under the same plasma power. Since the PR etching rate decreased rapidly with decreasing the working pressure, IGZO/PR selectivity was favorable at low working pressure. This meant desirable results showing better selectivity at the lower working pressure with high IGZO dry-etching rates. The top images of Figure 6a–c show the microscopic patterns after photo-process, dry etch, and PR strip. The IGZO dry etch condition was performed at the 75% HCl composition ratio and a bias power of 100 W under the working pressure of 3 mTorr. As shown in Figure 6, the 5 μm and 10 μm line widths were measured from the full width at half maximum in the two-dimensional depth profiles (bottom) of the PR and the IGZO at the same positions on the red-dotted line of the microscopic pattern images (top) by the laser microscope. The line widths of the 5 μm pattern after photo-process, dry etch, and PR strip were 4.8, 4.4, and 5.1 μm, respectively. The line widths of the 10 μm pattern after photo-process, dry etch, and PR strip were 9.8, 9.5, and 10.2 μm, respectively. The difference between the PR photo-patterned line width and the IGZO dry-etched patterned line width was 0.3 to 0.4 μm. This implies that excellent anisotropic etching on the IGZO without etching skew was carried out under this ICP-RIE etching condition. FIB-TEM analysis was performed to observe the cross-section of the etched pattern of IGZO. As shown in Figure 7a, the IGZO-etched profile exhibited an excellent taper angle of 6 degrees. This taper-etching profile was thought to be formed during the IGZO etching process, accompanied by the sidewall consumption of PR under ICP-RIE [29]. Figure 7b shows a schematic diagram of the taper etching mechanism of the IGZO film under ICP-RIE mode. This dry etching process could improve pattern resolution and bring excellent taper angle by improving the disadvantages of isotropic wet etching, such as undercut and pattern skew [22]. From the results of line width obtained from the pattern depth profile measurement by laser microscope and IGZO etch profile obtained from TEM, we were able to confirm that the IGZO-etched pattern with no undercut, etching skew-free, and excellent taper angle was obtained from anisotropic dry-etching of ICP-RIE.

**Figure 8:**
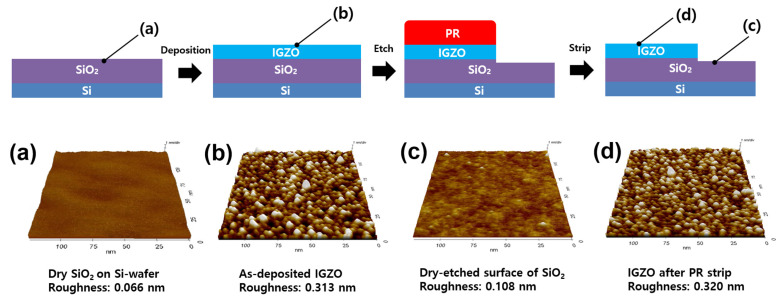
AFM images containing roughness of the top surfaces of (**a**) dry SiO_2_ on Si wafer, (**b**) as-deposited pristine IGZO, (**c**) dry-etched SiO_2_ by ICP-RIE, and (**d**) IGZO after PR strip.

**Figure 9:**
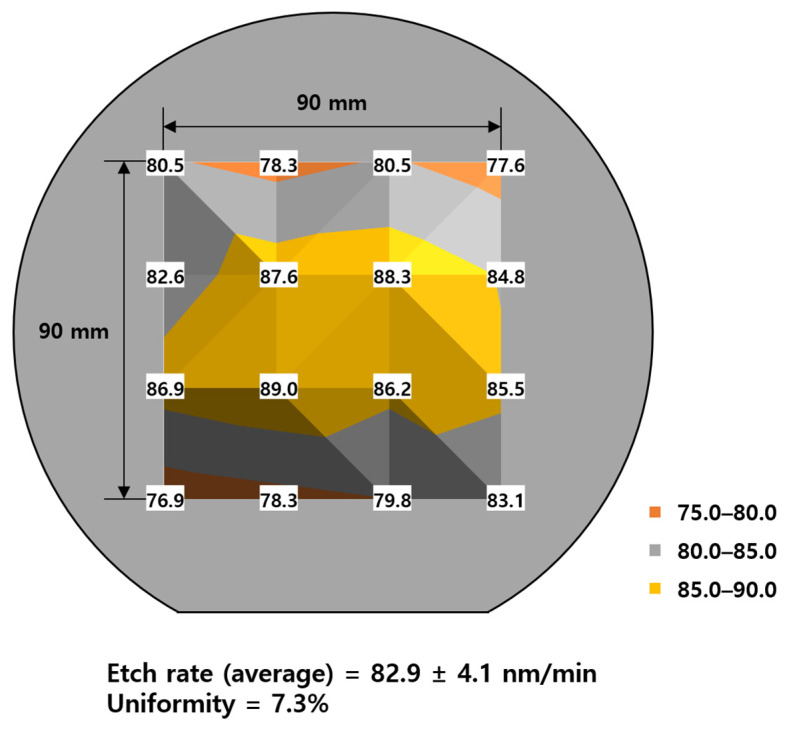
Etch rates and etch uniformity of IGZO film on a 6-inch wafer under optimal etching conditions such as plasma power of 100 W, process pressure of 3 mTorr, and HCl ratio of 75%.

On the other hand, AFM analysis was performed on the IGZO films before and after the etching process to understand the influence of the ICP dry etching on the surface of the SiO_2_ underneath the IGZO. Since the surface morphology of the IGZO and SiO_2_ may affect the electrical transfer characteristics in the TFT, it is essential to identify the surface roughness of the IGZO film after etching. Figure 8 shows the top-surface morphologies of dry SiO_2_ on Si wafer, as-deposited pristine IGZO, dry-etched SiO_2_ by ICP-RIE, and IGZO after PR strip. The dry SiO_2_ layer on the Si wafer showed an extremely smooth surface with a root mean square (RMS) roughness of 0.066 nm. The as-deposited pristine IGZO film had quite a rough surface of 0.313 nm. Since the IGZO layer was over-etched by ICP-RIE, the surface of the SiO_2_ layer should be slightly dry-etched, which exhibited a roughness of 0.108 nm. It could be said that the dry-etched surface roughness value was also good enough because it is comparable to the bare, dry SiO_2_ surface on the wafer. Etch rates and etching uniformity of IGZO film on a 6-inch wafer were measured under the optimal etching condition (plasma power of 100 W, process pressure of 3 mTorr, and HCl ratio of 75%). As shown in Figure 9, the average etch rate and etching uniformity of IGZO film on a 6-inch wafer were 82.9 ± 4.1 nm/min and 7.3%, respectively.

**Figure 10:**
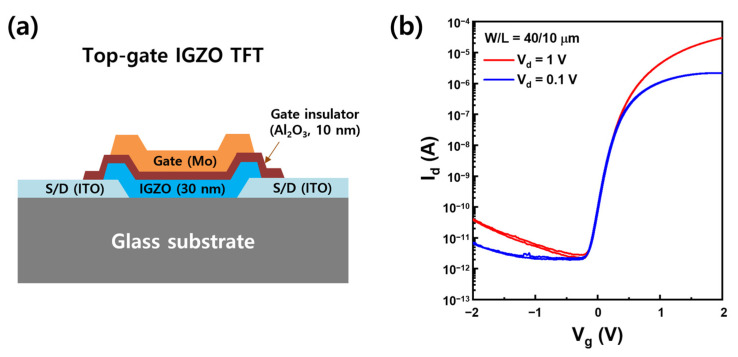
(**a**) Schematic cross-sectional view of top-gate IGZO TFT and (**b**) I_d_-V_g_ characteristics of IGZO TFT patterned by HCl/Ar-based ICP-RIE dry etching of the active layer.

A top-gate TFT was fabricated by patterning IGZO of 30 nm at the ICP-RIE dry-etching condition of 75% HCl composition ratio and a bias power of 100 W under the working pressure of 3 mTorr. Figure 10a shows the schematic cross-section of the top-gate IGZO TFT. Figure 10b shows the excellent I_d_-V_g_ characteristics of the IGZO TFT, exhibiting field effect mobility of 8.32 cm^2^/Vs, threshold voltage of 0.43 V, and subthreshold slope of 0.89 V/dec. The IGZO TFT showed an excellent subthreshold slope with no hysteresis in the transfer curve. It means that the IGZO channel interface was not almost affected by plasma exposure during the harsh ICP-RIE etching [30,31]. As a result, this HCl/Ar-based ICP-RIE dry-etching proved to be an efficient and useful method for accurately patterning the IGZO active layers in TFTs for high-resolution display.

## 5. Conclusions

The dry etching characteristics of the IGZO thin film in the ICP-RIE mode were investigated using the HCl-Ar etching gas mixture. The HCl gas composition ratio, bias power, and process chamber working pressure were essential parameters affecting the etch rate of the IGZO film. The increase in the HCl composition ratio increased the IGZO etch rate. The highest etch rate was found in chamber pressure of 3 mTorr at an HCl composition ratio of 75% (HCl:Ar = 30 sccm:10 sccm). When the working pressure exceeds the critical point of 3 mTorr, the etching rate might decrease due to a decrease in the mean free path of active species in the HCl-Ar plasma. Therefore, dry etching of IGZO films was successfully performed with a fast etch rate, excellent taper-etching profile, and smooth etched surface under a moderate RIE power of 100 W with a strong ICP source power of 500 W. As a result, we demonstrated top-gate IGZO TFT with excellent electrical performance by using the ICP-RIE mode to form a fine IGZO film pattern with minimizing etching damage on the PR surface as well as etching skew of the fine IGZO pattern due to strong anisotropic etching behavior.

## Data Availability

The original contributions presented in the study are included in the article/Supplementary material, further inquiries can be directed to the corresponding authors.

## Supplementary Materials

The following supporting information can be downloaded at: https://www.mdpi.com/article/10.3390/ma17246241/s1, Figure S1: Etch rates of the SiO2 films as a function of the HCl composition ratio according to the chamber working pressure at the ICP source power of 500 W and bias power of 100 W. Figure S2: Etch rates of the photoresist films as a function of the HCl composition ratio according to the chamber working pressure at the ICP source power of 500 W and bias power of 100 W.
