# Supplementary material for: Dry Etching Characteristics of InGaZnO Thin Films Under Inductively Coupled Plasma–Reactive-Ion Etching with Hydrochloride and Argon Gas Mixture
Source: Materials (Basel). 2024 Dec 20;17(24):6241. doi: 10.3390/ma17246241 (PMC11676547; doi:10.3390/ma17246241)
Supplement: Supplementary file 1 [file materials-17-06241-s001.zip › materials-3295943-supplementary.pdf]

Supplementary Information

Dry etching characteristics of InGaZnO thin films under inductively coupled plasma-reactive ion etching with hydrochloride and argon gas mixture

Changyong Oh[1], Myeong Woo Ju[2,3], Hojun Jeong[4], Jun Ho Song[4], Bo Sung Kim[2,3,4,*], Dae Gyu Lee[5,*], and ChoongHo Cho[5]

[1] DRAM Process Architecture Team, Samsung Electronics, Hwaseong-si, Gyeonggi-do, 18448, Republic of Korea

[2] Department of Applied Physics, Korea University, Sejong, 30019, Republic of Korea

[3] E·ICT-Culture·Sports Track, Korea University, Sejong 30019, Republic of Korea

[4] Division of Display and Semiconductor Physics, Korea University, Sejong, 30019, Republic of Korea

[5] Department of Computer Information Science, Korea University, Sejong, 30019, Republic of Korea

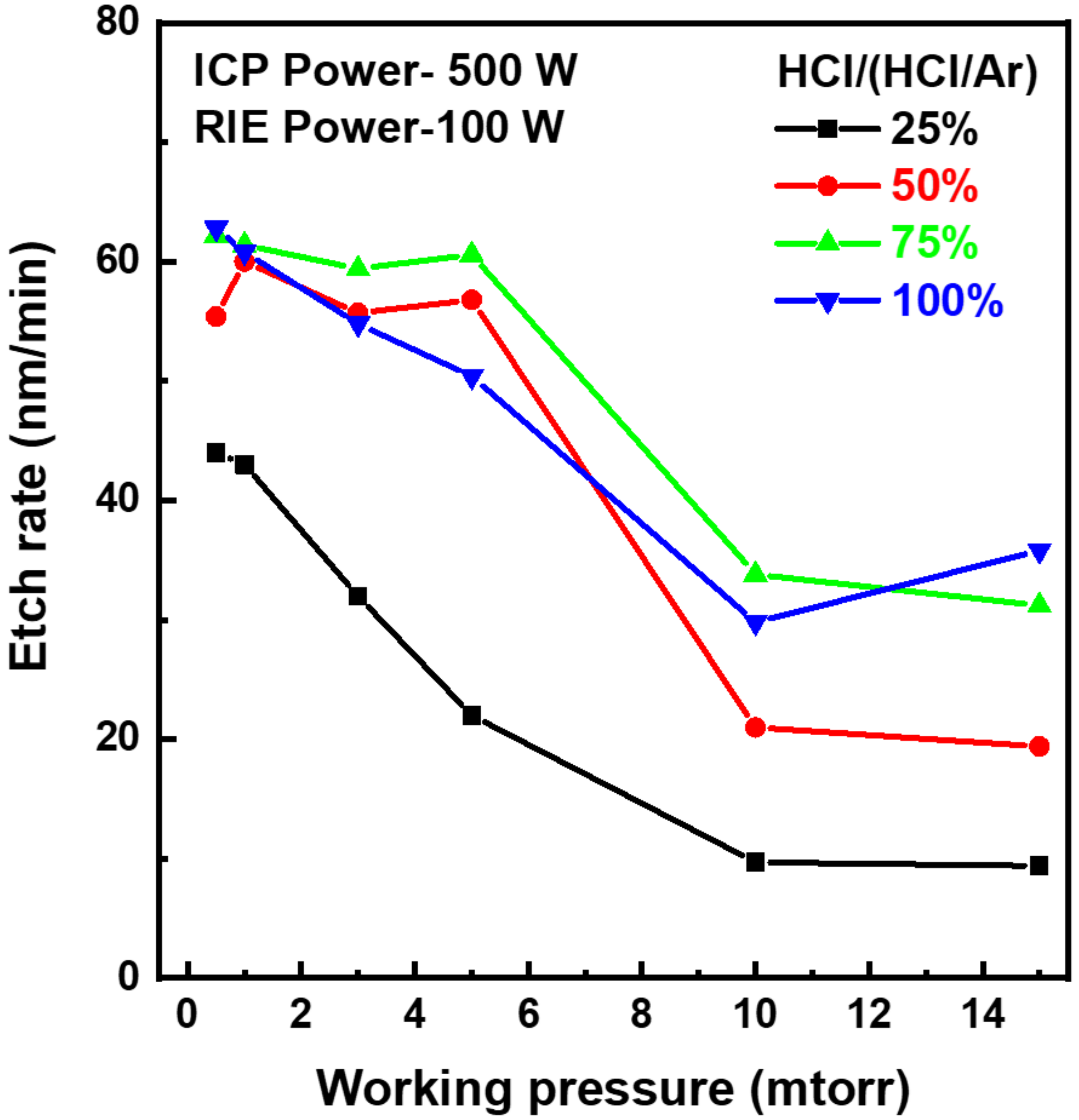

Figure S1. Etch rates of the SiO_2 films as a function of the HCl composition ratio according to the chamber working pressure at the ICP source power of 500 W and bias power of 100 W.

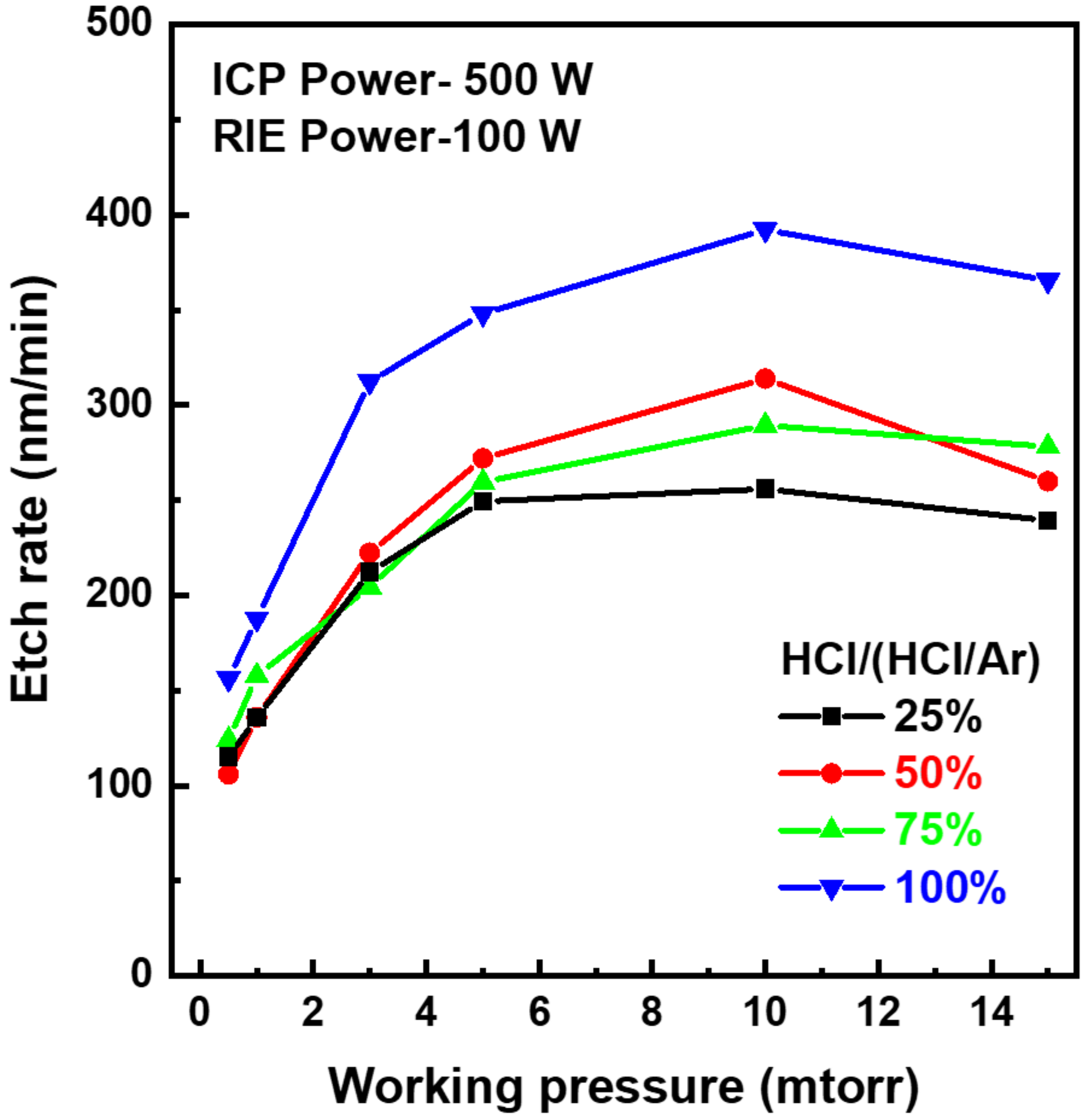

Figure S2. Etch rates of the photoresist films as a function of the HCl composition ratio according to the chamber working pressure at the ICP source power of 500 W and bias power of 100 W.
